# The Role of Water Content of Deep Eutectic Solvent Ethaline in the Anodic Process of Gold Electrode

**DOI:** 10.3390/molecules28052300

**Published:** 2023-03-01

**Authors:** Jie-Du Wu, Yu Ding, Feng Zhu, Yu Gu, Wei-Wei Wang, Lan Sun, Bing-Wei Mao, Jia-Wei Yan

**Affiliations:** 1 State Key Laboratory of Physical Chemistry of Solid Surfaces, Department of Chemistry, College of Chemistry and Chemical Engineering, Xiamen University, Xiamen 361005, China; 2 College of Chemistry and Bioengineering, Yichun University, Yichun 336000, China

**Keywords:** deep eutectic solvents, ethaline, water content, electrochemical etching, anodic dissolution, passivation

## Abstract

Traditional coupling of ligands for gold wet etching makes large-scale applications problematic. Deep eutectic solvents (DESs) are a new class of environment-friendly solvents, which could possibly overcome the shortcomings. In this work, the effect of water content on the Au anodic process in DES ethaline was investigated by combining linear sweep voltammetry (LSV) and electrochemical impedance spectroscopy (EIS). Meanwhile, we employed atomic force microscopy (AFM) to image the evolution of the surface morphology of the Au electrode during its dissolution and passivation process. The obtained AFM data help to explain the observations about the effect of water content on the Au anodic process from the microscopic perspective. High water contents make the occurrence of anodic dissolution of gold at higher potential, but enhances the rate of the electron transfer and gold dissolution. AFM results reveal the occurrence of massive exfoliation, which confirms that the gold dissolution reaction is more violent in ethaline with higher water contents. In addition, AFM results illustrate that the passive film and its average roughness could be tailored by changing the water content of ethaline.

## 1. Introduction

Wet etching of gold is an industrial technique for fabricating microstructures, which play a vital role in electronic and microelectromechanical systems [1]. Owing to adjustable current distribution and mass transport, electrochemical etching provides a better control in the etching profile of the gold substrate, thus attracting considerable attention in microfabrication [2,3,4,5]. Traditionally, thiourea- and cyanide-based ligands have been used in etching processes for complexation, but their toxicity cannot be bypassed [6,7,8]. Iodine-iodide etchant has been considered as an alternative [9,10], however, the problem of iodine vapor with quite a poisonousness is still worth considering [11,12]. Therefore, it is significant to develop novel etchants to alleviate safety concerns.

Deep eutectic solvents (DESs) are usually made up of an organic salt and a hydrogen bond donor (HBD) molecule in a specific molar ratio, leading to a eutectic melting point that is much lower than the melting points of the individual constituents [13,14,15]. In addition to their attractive properties, such as being nonflammable, low vapor pressure, as well as good biocompatibility and biodegradation, DESs can be produced at low costs [16,17]. The unique physicochemical properties of DESs are associated with the nature and strength of the intermolecular interactions, primarily hydrogen bonds [15,18,19,20]. As a consequence, as a new type of “green” environment-friendly solvents, DESs have already been applied to the fields of electrochemistry [16,21,22,23], electroanalysis [24,25,26], catalysis [27,28,29] and biotransformation [30,31].

The anodic processes of nine metals in ethaline (a mixture of choline chloride (ChCl) and ethylene glycol (EG) in a molar ratio of 1:2) were exhibited by Abbott *et al.*, and they demonstrated that there were anodic dissolution and passivation processes in high anodic polarization region [32]. In our previous work, the effect of HBD molecules on the anodic dissolution and passivation process of the Au electrode, as well as the electrochemical behaviors in the double-layer region, has been investigated [33]. In this respect, cyanide-, thiourea-, and iodide-free DESs are promising media for electrochemical etching owing to their high thermal and chemical stabilities as well as non-volatile features, which negate the emission of volatile organic compounds during the etching process.

Many DESs including ethaline tend to absorb water when exposed to the ambient atmosphere [34]. Water molecules could change the interactions between the two components of DES, e.g., HBD-anion interaction, and alter their physicochemical properties [35]. Particularly, the addition of controlled amounts of water to highly viscous DESs has emerged as an attractive route to enhance conductivity, fluidity, and solvation [36]. Some studies showed that DESs possess distinct molecular-scale nanostructure both in the bulk liquid [37] and at interfaces [38,39,40]. The presence of water, in both small and large quantities, could influence the nanostructure in DESs since the liquid components must re-arrange to accommodate the solute. Kaur et al. probed the bulk nanostructure of ethaline as a function of water composition by MD simulations, demonstrating that a transition from an ethaline-like structure to a water-like structure between 25.5 and 40.6 wt% of water [41]. The ethaline-water system is best described as an aqueous solution of ChCl and EG at higher water content. Similarly, the higher concentrations of water (42 wt%) significantly disrupted the native liquid nanostructure of reline (a mixture of ChCl and urea in a molar ratio of 1:2) [42].

The interfacial structures of three DESs (reline, ethaline, glyceline) have also been explored by using atomic force microscopy (AFM)-based force curve measurements, revealing that the interfacial nanostructure increases with the addition of water up to ~40 wt%, and then decreases at open circuit potential [38]. It was presented that the involvement of water molecules in the hydrogen bond network of the DESs benefits the formation of layer packaging. Theoretically, the electrosorption and distribution of water at the electrified graphene interface in reline was revealed by employing atomistic molecular dynamics [43]. At moderate polarization, the water molecules are depleted from the negatively charged electrode and accumulated at the positively charged electrode due to the interactions between water and chloride anion. At high polarization, water accumulates at both electrodes due to the electrostatic interactions.

The effect of water on the electrochemical reactions in DESs has been investigated, especially their application in electrodeposition. It was found that the addition of water was beneficial the mass transport [44], and could result in the change of the structure of the electrical double layer [38,45]. The addition of water into ethaline enhances transport and facilitates higher deposition rates for Cu electrodeposition [46]. In addition, an increase in water content alters not only the interaction between liquid components, but also the structure of Ni^2+^ complexes and thus leads to the change of reduction potential of Ni^2+^ in Ni electrodeposition [47]. 

In this work, the effect of water content on the Au anodic process in ethaline was investigated by combining linear sweep voltammetry (LSV) and electrochemical impedance spectroscopy (EIS). Meanwhile, we employed AFM to image the evolution of the surface morphology of the Au electrode during its dissolution and passivation process. The obtained AFM data help to explain observations about the influence of water content on the Au anodic process from the microscopic perspective. Furthermore, a scanning electron microscope (SEM) was employed to explore the overview of the evolution of gold surface on an anodic process.

## 2. Results and Discussion

**Figure 1:**
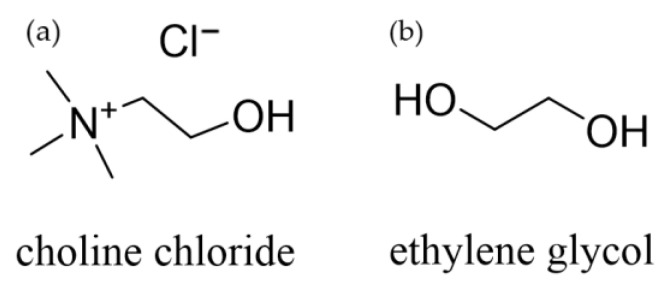
Chemical structures of ethaline components, (**a**) choline chloride (ChCl); (**b**) ethylene glycol (EG).

**Figure 2:**
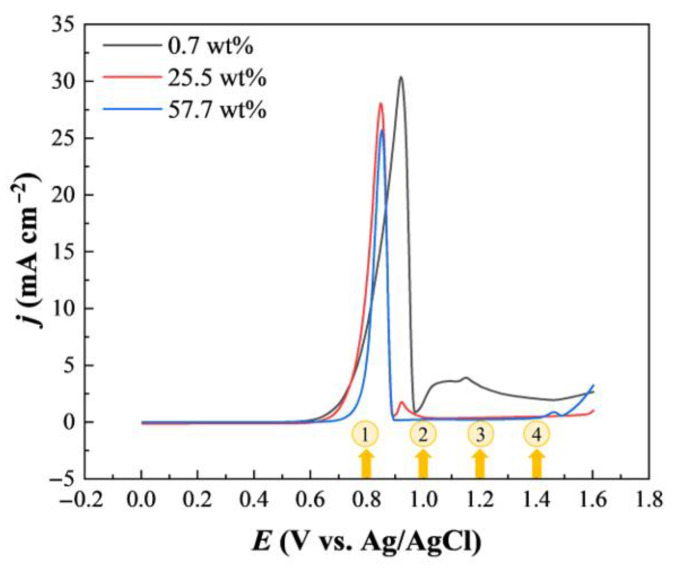
Linear sweep voltammograms of Au electrode in ethaline with different water contents (black line: 0.7 wt%; red line: 25.5 wt%; blue line: 57.7 wt%). Scan rate: 5 mV/s. Yellow circled 1, 2, 3, 4 represent 0.8 V, 1 V, 1.2 V, 1.4 V, respectively.

The chemical structures of ethaline components, choline chloride and ethylene glycol, were depicted in Figure 1. We chose three representative water contents 0.7 wt%, 25.5 wt%, and 57.7 wt% to investigate the effect of water content on the Au anodic process. As shown in Table 1, the corresponding molar ratios of water to ethaline are 0.1:1, 5:1, and 20:1, respectively. Figure 2 presents the linear sweep voltammograms of Au electrodes in ethaline with different water contents at a sweep rate of 5 mV/s. The voltammetric responses demonstrate an oxidation peak with a sharp increase in current density on the anodic process under the three water contents. Normally, the anodic limit of potential was cut off at the beginning of this current peak due to the rapid rising of current, which was regarded as the electrochemical window of the Au electrode in DESs [48,49,50]. However, with the further positive shift of the potential, the current reached its peak value and then decreased sharply. Subsequently, the current leveled off and was almost equal to the baseline, indicating that a passivation layer formed on the electrode surface.

Ethaline with different water content exhibited different electrochemical behaviors on the Au electrode. In the active region, for “pure” ethaline (0.7 wt%, black line in Figure 2), the anodic current began to increase at ~0.6 V, suggesting that the dissolution of gold happened. When water was added to ethaline, the onset potential gradually shifted to a higher potential. For ethaline with 57.7 wt% water, the potential shifted to ~0.7 V. In our previous study, we found that chloride anions could strongly adsorb on the Au electrode surface by forming an Au-Cl bond at a positively charged surface [51]. In addition, with the positive shift of the potential, a disorder-order phase transition of Cl^−^ adlayer was observed [51,52]. This indicates that gold anodic dissolution in ethaline is mainly induced by chloride ions. The increase in water content decreases the concentration of chloride ions in ethaline. Based on the LSV results, the lower concentration of chloride ions accounts for the positive shift of the potential for the anodic dissolution of gold.

In the active-passive region, for “pure” ethaline (0.7 wt%), the current peaks at ~0.9 V and then decreases sharply. The region of negative slope corresponds to the increased rate of passive film formation, where the formation rate is much larger than the active dissolution rate. It can be seen that the current peak shifted to lower potentials and the peak value decreased when the water content increased. Besides the change in chloride concentration at the interface, this can be concerned with the improved conductivity of the electrolyte as the increase of water content.

Interestingly, in the passive region, the current increases again for “pure” ethaline (0.7 wt%). It can be observed that the relatively obvious current exists within the whole region, indicating that the formed passive film may be not stable and dense enough to completely block the electrode surface. As the water content increases to 25.5 wt%, a small current peak appears at around 0.92 V and rapidly decreases. With the increase of water content to 57.7 wt%, the current levels off and is almost equal to the baseline within the passive region. It indicates that the passive film formed is much denser and completely blocks the electrode surface, thus hindering the continuation of the reaction.

**Figure 3:**
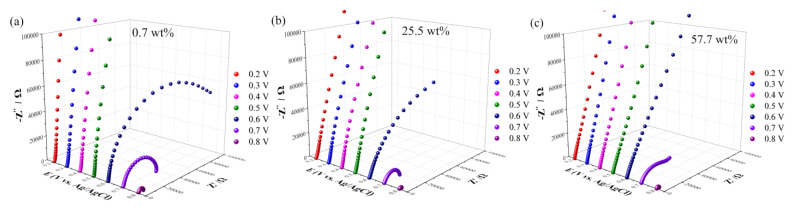
(**a**–**c**) Electrochemical impedance spectra of Au electrode in ethaline with different water contents at various potentials: (**a**) 0.7 wt%, (**b**) 25.5 wt%, (**c**) 57.7 wt%.AC amplitude: 10 mV; frequency range: 10^5^–1 Hz.

Electrochemical impedance spectroscopy, as a significant method for understanding the interfacial behavior, can be exploited for investigating electrochemical dissolution and passivation of the metal electrodes [53,54]. Compared with mass transport, the information about charge transfer at the electrolyte/electrode interface is present in the higher frequency region. The electrochemical impedance spectra of Au electrodes in ethaline with different water contents at various potentials are shown in Figure 3. The potential was shifted in the positive direction during the measurements. The impedance spectra for all three water contents present typical double-layer behavior from 0.2 to 0.5 V [50,55,56]. A single semicircle corresponding to the process of electron transfer was observed at 0.6 V, which should be due to the anodic dissolution of gold in “pure” ethaline (0.7 wt%). As the potential is shifted positively, the semicircle lessens its width indicating a faster electron transfer process that is caused by a positive polarization. As for ethaline with 57.7 wt% water content, the double-layer behavior remains at 0.6 V. A semicircle begins to appear at 0.7 V, demonstrating that the occurrence of anodic dissolution of gold lags compared with “pure” ethaline, which is consistent with the LSV results.

Figure S2 presents the Nyquist plots and the corresponding fitted curves obtained in ethaline with the three water contents. The equivalent circuit is shown in Figure S1, and the fitting data are listed in Table S1 (Supplementary Materials). A distinct semicircle is present at 0.8 V for all three systems, and the radius of the semicircle corresponds to the charge transfer resistance (R_ct_). By comparing the fitting values of R_ct_, we can see that the charge transfer resistance for “pure” ethaline exhibited the largest value of 5088.0 Ω. As for 25.5 wt% and 57.7 wt%, the charge transfer resistance decreased to 2182.6 and 2802.5 Ω, highlighting the important role of the water molecules in enhancing the electron transfer and gold dissolution at the interface. The water duality properties act as HBA and HBD. Significantly, water can interact with both HBA and HBD of ethaline and then break down the hydrogen bond interactions between ChCl and EG by forming a multi-hydrogen bond with the hydrogen bond donor [17]. The addition of water to ethaline alter the physicochemical properties and it would define the anodic process. The pH value and viscosity decrease as the water content increases [57,58,59]. The viscosity of the solvents plays an important role in their electrochemical behavior [47]. When the water concentration increases from 0.7 to 57.7 wt%, with a drastic decrease in the viscosity, would influence the mass transport. As can be seen from the LSV curves (Figure 2), the current density of 25.5 wt% (red line) is higher than 0.7 wt% (black line) at 0.8V, implying the faster reaction rate, and it can be attributed to the effect of physicochemical properties caused by water. However, with the increase of water content to 57.7 wt% (blue line), the current density decreased distinctly. The concentration of chloride anions in DES is reduced when adding water. Chloride anions play an important role in the anodic process of Au by forming an Au-Cl bond to induce the dissolution of gold and further impact the formation of the passive film. The reaction rate originates as a result of two factors, i.e., the viscosity of the electrolyte and the concentration of chloride anion. As a consequence, the reaction rate of 57.7 wt% is lower than 25.5 wt%, which is coincident with the variation of charge transfer resistance (R_ct_) of EIS results.

**Figure 4:**
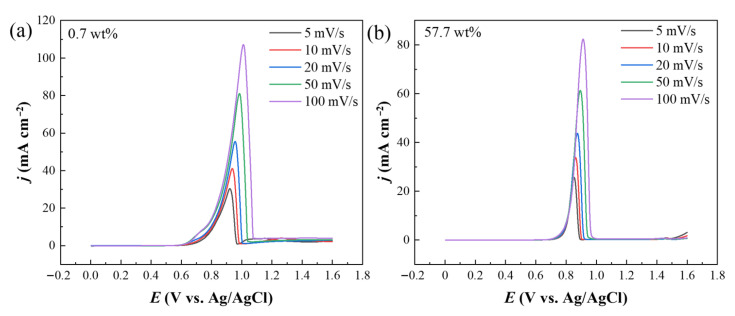
(**a**,**b**) Linear sweep voltammograms of Au electrode in ethaline with different water contents: (**a**) 0.7 wt%, (**b**) 57.7 wt%. Scan rates are 5, 10, 20, 50, and 100 mV/s.

Taking into account the results of LSV and EIS, the water content of ethaline shows a significant influence on the Au electrode. Further, we scrutinize the anodic dissolution process in ethaline with water contents of 0.7 and 57.7 wt% by employing various scan rates. As can be clearly seen from Figure 4, when the scan rate increases from 5 to 100 mV/s, the peak current density increases gradually and the peak potential moves positively. If the oxidation peak current density is proportional to the square root of the scan rate, the reaction can be considered to be controlled by the diffusion of solutes from the bulk to the electrode. The relationship between the oxidation peak current density and the scan rate is evaluated by plotting the values of the former against the square root of the latter (Figure S3a,b). The correlation coefficients of the plots in Figure S3a,b are 0.994 and 0.989 for 0.7 and 57.7 wt%, respectively, which present a little deviation from the unity, i.e., the anodic process is not just controlled by the diffusion. The diffusion control of the anodic process can also be indicated by the angular coefficient of the logarithmic dependence of the peak current density (ip) on the scan rate (v): Xv $=\left(\frac{\Delta \log ip}{\Delta \log v}\right)$ [60]. Figure S3c,d demonstrate that the curves for the two water contents are linear, however, the coefficient Xv of 0.7 and 57.7 wt% is 0.424 and 0.388, respectively, which is smaller than 0.5. Thus, it is reasonable to assume that besides diffusion, extra process is involved in the gold dissolution in DESs, which shows the complexity of Au anodic corrosion in ethaline.

The influence of water content on the anodic process of the gold electrode in ethaline should be closely related to the water-induced change of interfacial structure. Water-induced alteration of native DES nanostructure at the solid-liquid interface has been investigated in the literature. Hammond et al. reported that water molecules dramatically altered the interfacial structure between DESs and platinum at high hydration levels [38], which is consistent with the observation of Elbourne et al. [39]. It showed that the interface interaction is obviously different when the property of the electrolyte gradually changes from ethaline-like to water-like. Particularly, compared with aqueous electrolyte, the anodic dissolution of gold electrode occurs at the lower potential in ethaline, and the passive film formed subsequently.

To understand the evolution of gold surface morphology during the anodic dissolution process, AFM measurements were conducted for Au(111) single crystal electrode with the flat and well-defined surface after holding at different potentials in ethaline with 0.7 and 57.7 wt% water contents. Specifically, the Au(111) electrode was freshly prepared by electrochemical polishing and hydrogen flame annealing to obtain a well-defined surface. Next, the Au(111) electrode was immersed in ethaline and kept at various potentials for 3 min. The electrode was then taken out and washed twice in pure ethyl alcohol to remove the residual ethaline, and further rinsed with ultrapure water thoroughly.

**Figure 5:**
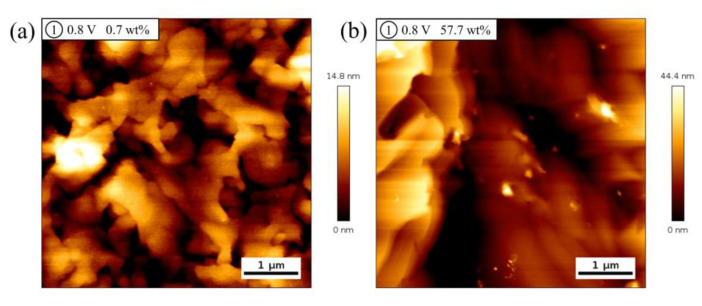
(**a,b**) AFM images of Au(111) electrode surface after holding at 0.8 V for 3 min in ethaline with water contents 0.7 wt% (**a**) and 57.7 wt% (**b**), respectively.

The freshly prepared Au(111) surface was first probed by AFM in tapping mode (Figure S4), and the average roughness was measured to be around 310 pm across an area of 5 um by 5 um. Hence, the Au(111) surface is smooth enough for further observation of the anodic dissolution process. Figure 5 shows typical AFM images of the Au(111) surface after holding at 0.8 V for 3 min in ethaline with 0.7 wt% and 57.7 wt% water contents. It can be seen from the AFM images that surface etching occurs due to the gold anodic dissolution, but the surface topographies after etching in the two water contents are quite distinct from each other, indicating that the etching behavior obviously depends on the water content. As illustrated in Figure 5a, the morphology is composed of relatively disordered and rugged structures. When water content increases to 57.7 wt%, the morphology is dominated by layer-shaped structure (Figure 5b), which suggests the occurrence of massive exfoliation, indicating that the gold dissolution reaction is more violent in ethaline with 57.7 wt% water contents.

On the whole, the anodic dissolution behavior of gold in ethaline is more violent than that in an aqueous electrolyte containing chlorine [61] and ionic liquid [62], which may be due to the high concentration of chloride ions in ethaline. The addition of water promotes the anodic dissolution of gold, which is consistent with the observed difference in the reaction rates. Based on the above results, we propose the possible chemical equation for the characteristic electrochemical oxidation of gold electrodes in ethaline:

Au + Cl^−^ = (AuCl^−^)_ad_

(AuCl^−^)_ad_ + Cl^−^ = (AuCl_2_^−^)_ad_ + e^−^

(AuCl_2_^−^)_ad_ = AuCl_2_^−^ (dissolution)

(AuCl_2_^−^)_ad_ + 2Cl^−^ = (AuCl_4_^−^)_ad_ + 2e^−^

(AuCl_4_^−^)_ad_ = AuCl_4_^−^ (dissolution)

**Figure 6:**
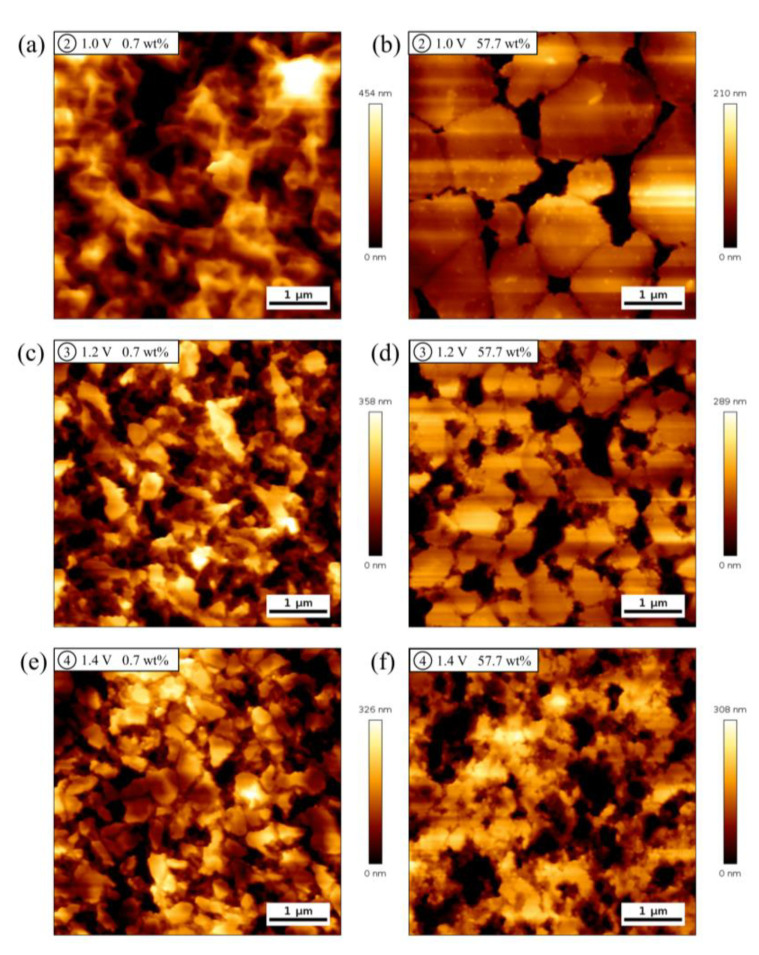
(**a**−**f**) AFM images of Au(111) electrode surface after holding at different potentials for 3 min in ethaline with water contents 0.7 wt% (**a**,**c**,**e**) and 57.7 wt% (**b**,**d**,**f**). (**a**,**b**) 1 V; (**c**,**d**) 1.2 V; (**e**,**f**) 1.4 V.

Further, to directly visualize the influence of water content on the formation of passive film on the gold surface from a microscopic perspective, AFM measurements were performed to image the morphological changes. Typical AFM images of the Au(111) surface were acquired after Au(111) electrode was held at 1.0, 1.2, and 1.4 V for 3 min in ethaline with water contents 0.7 wt% and 57.7 wt%, respectively (Figure 6a–f). It can be clearly observed from Figure 6a that the morphology becomes more fragmented, indicating that the passive film generated at this potential is not dense enough. This phenomenon is consistent with the change of current in the LSV curve (black line of Figure 2). The current decreases and then increases again in this potential region. Interestingly, when the water content increased to 57.7 wt% (Figure 6b), although the gold surface fluctuation is larger than two hundred nanometers, the morphology is dominated with island-like domains, indicating that the passive film formed in this case may be relatively compact. Correspondingly, the current in the LSV curve of Figure 2 does not increase in the passive region. It is worth noting that in an aqueous solution the current in the LSV curve of gold anodic process will not fall to the same level as the baseline, indicating that the passive film formed in an aqueous solution is not dense, in comparison to the observations in DES [61,63,64]. In halogen ion-modified IL, no formation of the compact passive film was observed [62].

The morphology of the passive film undergoes further modifications as the potential is shifted positively to 1.2 V. Although the surface fluctuation varies slightly, more massive structures appear in the morphology, suggesting that the passive film is improved (Figure 6c). Compared with “pure” ethaline, the number of island-like domains increases and the corresponding size decreases at 1.2 V for ethaline with 57.7 wt% water content (Figure 6d).

When the potential is further moved to 1.4 V, it can be seen that more massive structures form on the gold surface in ethaline with 0.7 wt% water content (Figure 6e). Correspondingly, there is still some residual current in the LSV curve (Figure 2), indicating that the passive film does not completely block the electrode surface. In contrast, when the water content is 57.7 wt%, the larger island-like domains appearing at 1.0 V are further broken into smaller pieces at 1.4 V (Figure 6f). As a result, the passive film began to decompose at the potential and the decomposition current can be clearly observed in the LSV curve as the potential is shifted positively (blue line of Figure 2).

To further understand the influence of water contents, statistical analyses of the average roughness of Au(111) electrode surface determined from the AFM images as a function of applied potentials are made, which are shown in Figure S5. For 0.7 wt% water content, the average roughness fluctuates with the positive shift of applied potentials. The roughness reaches the maximum value when the applied potential is 1.0 V, and then slightly decreases. However, for 57.7 wt% water content, the average roughness increases progressively with the positive shift of the potential. In the passive region, the roughness is lower than that of 0.7 wt% water content.

The above AFM results illustrate that the topography of the gold surface is dependent on the applied potentials, and passive film and average roughness could be tailored by changing the water content of ethaline. It is worth recalling that in a 0.1 M perchloric acid (HClO_4_) solution containing chloride anion, Ye et al. investigated the anodic dissolution process of Au(111) by using an in-situ scanning tunneling microscope [61]. They found that the dissolution of Au(111) anisotropically proceeds in a layer-by-layer mode. In terms of current density and morphology, the etching of gold surface is relatively mild in an aqueous solution [65,66]. In addition, in ionic liquids, Ueda et al. employed halide-modified gold electrodes to investigate the electrochemical oxidation of gold, finding that only iodine could have impacted the etching of gold [62]. In this choline chloride-based DES, the vigorous dissolution of gold electrodes and the influence of water contents are revealed, which show promising applications in the wet etching of the gold industry.

**Figure 7:**
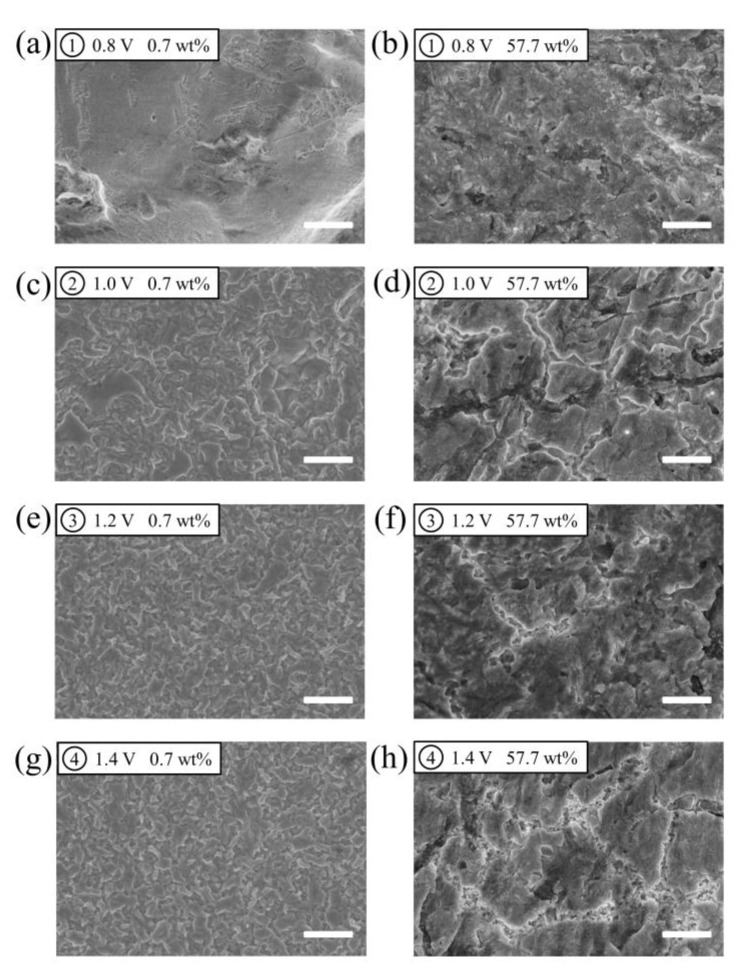
(**a**−**h**) SEM images of a gold foil after holding at different potentials for 3 min in ethaline with water contents 0.7 wt% (**a**,**c**,**e**,**g**) and 57.7 wt% (**b**,**d**,**f**,**h**). (**a**,**b**) 0.8 V; (**c**,**d**) 1.0 V; (**e**,**f**) 1.2 V; (**g**,**h**) 1.4 V. Scale bars are 2 μm.

Furthermore, SEM was employed to explore the overview of the evolution of gold surface on an anodic process. Figure 7 shows the SEM images of the surface of Au foil after holding at different potentials for 3 min in ethaline with water contents of 0.7 wt% and 57.7 wt%. Some scratches observed on the surface before the anodic dissolution were caused by the mechanical polishing of the electrode (Figure S6). A bulk dissolution of gold was observed after holding at 0.8 V. When the water content increases to 57.7 wt%, the morphology is more fractured. In the passive region, the passive film was formed on the surface of gold. The increase in water content alters the morphology of the film, which accords with the AFM results.

## 3. Materials and Methods

Choline chloride (Sigma-Aldrich, St. Louis, MO, USA, ≥98%) and ethylene glycol (Sigma-Aldrich, St. Louis, MO, USA, ≥99.8%) were mixed in a molar ratio of 1:2 and stirred at 333 K in a silicone oil bath till a homogeneous solution formed. Prior to the use, ethaline was vacuum-dried for one hour at 333 K to remove the absorbed water and oxygen in a glove box filled with Ar (99.999%). Water contents were determined by Karl Fischer Coulometer (Metrohm, Herisau, Switzerland, KF-831). Ethaline with different water contents was prepared via adding ultrapure water (Milli-Q, 18.2 MΩ·cm).

All the electrochemical measurements were performed with an Autolab potentiostat (Metrohm, Switzerland, PGSTAT128N) controlled by the Nova 2.1 software package. A polycrystalline Au disk electrode was employed as a working electrode. The counter electrode was a platinum wire wound in a ring. The reference electrode was Ag/AgCl electrode and a salt bridge was used to avoid the contamination of the electrolyte. The temperature of the electrolytes in the cell was maintained at 23 ± 2 °C. Before the experiment, the Au electrode was polished with alumina (0.05 um) and then the electrode was cleaned in an ultrasonic bath and copiously rinsed with ultrapure water for three times [67]. Finally, the electrode was dried with nitrogen gas flow. IR compensation was implemented during linear sweep voltammetry measurements to decrease the deviation caused by relatively large currents [68]. The degree of correction is 80%. Solution resistance R is determined using the intercept of the intersection point with the X-axis in Nyquist plots at high frequency in the double-layer region. Electrochemical impedance spectroscopy experiments were recorded from 100 kHz to 1 Hz with an amplitude of 10 mV. The impedance spectra were analyzed by fitting the parameters of the equivalent circuit (Figure S1) to the measured spectra by using the RelaxlS 3 software. To avoid the disturbance from the external electromagnetic field, a shielded box was used.

AFM experiments were performed by using a JPK NanoWizard Sense AFM instrument (Bruker, Heidelberg, Germany). OLTESPA commercial silicon cantilever tips (2 N/m, 70 kHz) with a typical ~7 nm radius at the end were employed. All images were recorded in tapping mode. Au(111) single crystal electrodes with flat surfaces were used in order to clarify the evolution of gold surface nanostructure due to the electrochemical etching in ethaline. Au(111) electrodes were prepared following a modification of Clavilier’s method [69,70,71]. The electrodes were subjected to electrochemical polishing and flame annealing in hydrogen followed by cooling under a nitrogen atmosphere prior to each experiment. The morphologies of gold foil (thickness 0.5 mm, 99.99%) with different applied potential were characterized by scanning electron microscope (SEM, Hitachi, Tokyo, Japan, SU4800).

## 4. Conclusions

In summary, the characteristic anodic process of gold in ethaline has been systematically investigated. Combining LSV, EIS, and AFM measurements, we found that the gold surface underwent dissolution and passivation during the positive shift of the potential in ethaline DES. The reaction was evaluated to not merely be diffusion controlled, involving an extra process, which displays the complexity of the gold anodic process in ethaline. Furthermore, water molecules play a key role in the potential-induced anodic process by changing the interaction between ethaline and gold surface. Combining EIS with AFM and SEM measurements, the gold dissolution reaction is more violent in ethaline as water contents increase. This might be ascribed to the fact that water molecules could destroy the hydrogen bond network of DES. Therefore, the structure of the electrical double layer changed, and water and components of ethaline are involved in the reaction at the interface. The topography of the gold surface is dependent on the applied potentials, and passive film and average roughness could be tailored by changing the water content of ethaline. 

Our experimental results are helpful for the understanding of the effect of a water molecule on electrochemical reaction in ethaline. What is more, the anodic behavior of gold is of fundamental importance to extraction, refining, electroetching, and electropolishing. The findings reported here provide new insights into the enlargement of wet etching of gold to DESs systems. We employed DES, as environment-friendly solvents, to possibly overcome the shortcomings of the traditional toxicity process. Besides, water also can be added to not only purposely reduce their problematically high viscosity and lower their high price but also adjustably regulate the morphology and degree of wet etching.

## Figures and Tables

**Table 1:** Compositions of ethaline-water mixtures investigated in the present work.

Water Weight Percentage	Water Mole Percentage	Molar Ratio of Water:Ethaline
0.7 wt%	3.4 mol%	0.1:1
25.5 wt%	62.5 mol%	5:1
57.7 wt%	87.0 mol%	20:1

## Data Availability

The data are included in the article.

## Supplementary Materials

The following supporting information can be downloaded at: https://www.mdpi.com/article/10.3390/molecules28052300/s1, Figure S1: The equivalent circuit used for fitting EIS; Figure S2: The Nyquist plots of Au electrode in ethaline with different water contents at 0.8 V; Figure S3: (a,b) The plots of the oxidation peak current density vs. the square root of the scan rate in ethaline. (c,d) Semerano plots for the Au electrode in ethaline. Water contents: 0.7 wt% (a,c) and 57.7 wt% (b,d); Figure S4: AFM image of freshly prepared Au(111) electrode surface; Figure S5: Average roughness of Au(111) electrode surface as a function of applied potentials. Water content: 0.7 wt% (black); 57.7 wt% (red); Figure S6: SEM image of a gold foil before anodic dissolution; Table S1: Fitted parameters of Nyquist plots at 0.8V in ethaline with different water contents.

## Sample Availability

Samples of the compounds are available from the authors.
