# Supplementary material for: The Role of Water Content of Deep Eutectic Solvent Ethaline in the Anodic Process of Gold Electrode
Source: Molecules. 2023 Mar 1;28(5):2300. doi: 10.3390/molecules28052300 (PMC10005209; doi:10.3390/molecules28052300)
Supplement: Supplementary file 1 [file molecules-28-02300-s001.zip › molecules-2229360-supplementary.pdf]

Supplementary Materials

The Role of Water Content of Deep Eutectic Solvent Ethaline in the Anodic Process of Gold Electrode

Jie-Du Wu [1], Yu Ding [1], Feng Zhu [2], Yu Gu [1], Wei-Wei Wang [1], Lan Sun [1], Bing-Wei Mao [1,*] and Jia-Wei Yan [1,*]

[1] State Key Laboratory of Physical Chemistry of Solid Surfaces, Department of Chemistry, College of Chemistry and Chemical Engineering, Xiamen University, Xiamen 361005, China
[2] College of Chemistry and Bioengineering, Yichun University, Yichun 336000, China
* Correspondence: bwmao@xmu.edu.cn (B.-W.M.), jwyan@xmu.edu.cn (J.-W.Y.)

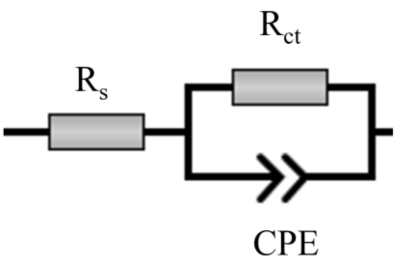

Figure S1. The equivalent circuit used for fitting EIS.

The obtained Nyquist plots were analyzed with RelaxIS software using the above equivalent circuit, where R_s is the solution resistance, R_{ct} is the charge transfer resistance, and CPE is the constant phase element.

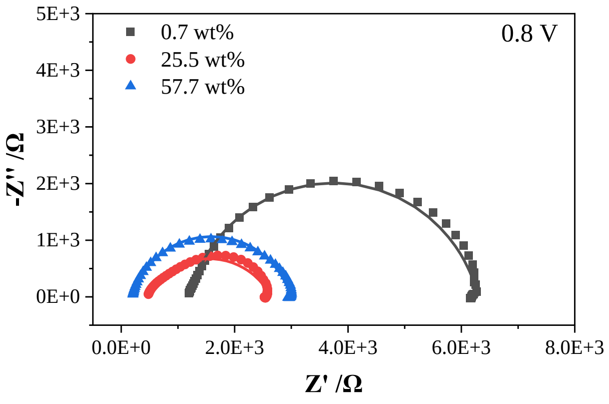

Figure S2. The Nyquist plots of Au electrode in ethaline with different water contents at 0.8 V.

Table S1. Fitted parameters of Nyquist plots at 0.8V in ethaline with different water contents.

Systems	Rs (Ω)	Rct (Ω)	CPE (Ω S^n)	n
0.7 wt%	1207.7	5088.0	2.042E-7	0.852
25.5 wt%	458.3	2182.6	1.445E-6	0.703
57.7 wt%	188.4	2802.5	3.139E-7	0.827

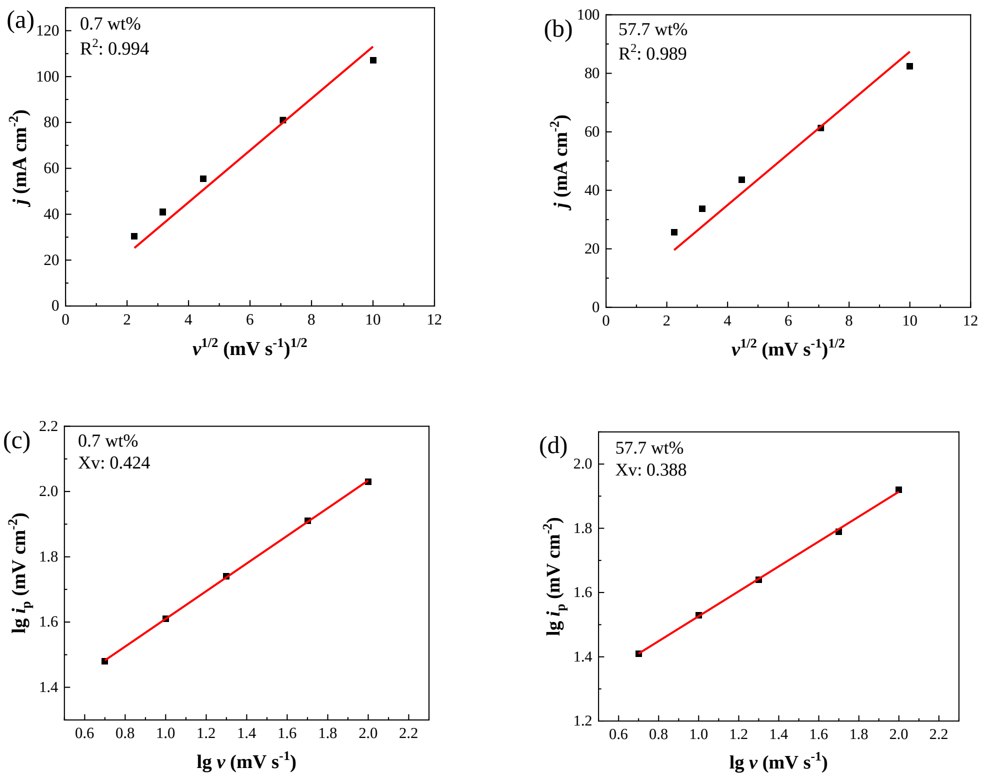

Figure S3. (a, b) The plots of the oxidation peak current density vs. the square root of the scan rate in ethaline. (c, d) Semerano plots for the Au electrode in ethaline. Water contents: 0.7 wt% (a, c) and 57.7 wt% (b, d).

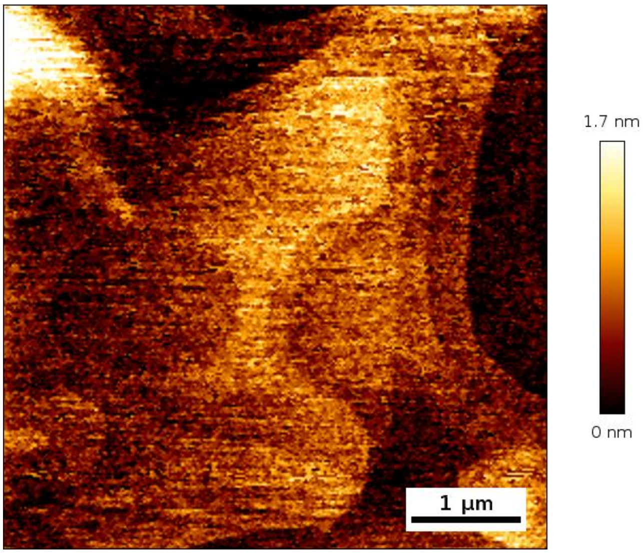

Figure S4. AFM image of freshly prepared Au(111) electrode surface.

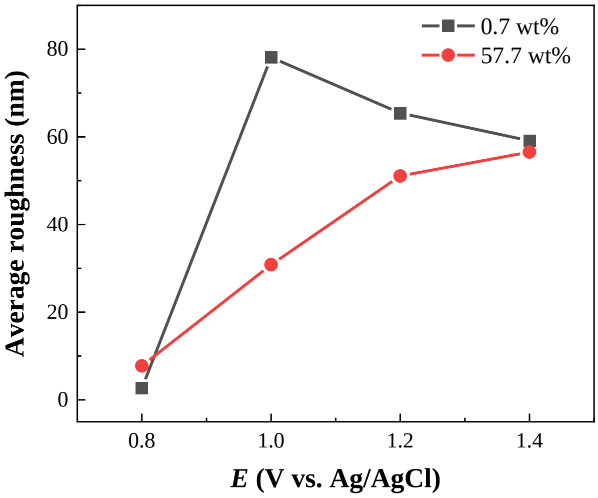

Figure S5. Average roughness of Au(111) electrode surface as a function of applied potentials. Water content: 0.7 wt% (black); 57.7 wt% (red).

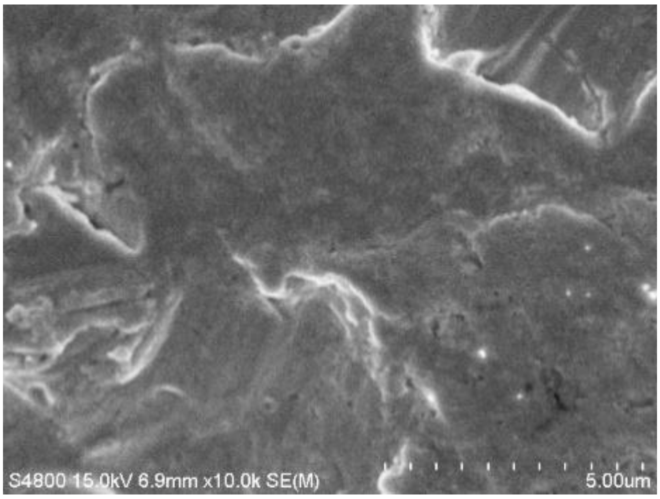

Figure S6. SEM image of a gold foil before anodic dissolution.
